# Patient-derived organoids as a platform for modeling a patient’s response to chemoradiotherapy in esophageal cancer

**DOI:** 10.1038/s41598-021-00706-8

**Published:** 2021-10-29

**Authors:** Tatiana A. Karakasheva, Joel T. Gabre, Uma M. Sachdeva, Ricardo Cruz-Acuña, Eric W. Lin, Maureen DeMarshall, Gary W. Falk, Gregory G. Ginsberg, Zhaohai Yang, Michele M. Kim, Eric S. Diffenderfer, Jason R. Pitarresi, Jinyang Li, Amanda B. Muir, Kathryn E. Hamilton, Hiroshi Nakagawa, Adam J. Bass, Anil K. Rustgi

**Affiliations:** 1 Division of Gastroenterology, Department of Medicine, Perelman School of Medicine, University of Pennsylvania, Philadelphia, PA USA; 2 Gastrointestinal Epithelium Modeling Program, Division of Gastroenterology, Hepatology and Nutrition, Children’s Hospital of Philadelphia, Philadelphia, PA USA; 3 Division of Thoracic Surgery, Massachusetts General Hospital, Boston, MA USA; 4 Department of Pathology and Laboratory Medicine, Perelman School of Medicine, University of Pennsylvania, Philadelphia, PA USA; 5 Division of Digestive and Liver Diseases, Department of Medicine and Herbert Irving Comprehensive Cancer Research Center, Columbia University Irving Medical Center, 1130 St. Nicholas Avenue, Suite 201, New York, NY 10032 USA; 6 Department of Radiation Oncology, Perelman School of Medicine, University of Pennsylvania, Philadelphia, PA USA; 7 Dana-Farber Cancer Institute, Harvard Medical School, Broad Institute, Boston, MA USA; 8 Herbert Irving Comprehensive Cancer Research Center, Columbia University Irving Medical Center, New York, NY USA; 9 Massachusetts General Hospital, Boston, MA USA

**Keywords:** Cancer, Translational research

## Abstract

3D patient-derived organoids (PDOs) have been utilized to evaluate potential therapies for patients with different cancers. However, the use of PDOs created from treatment-naive patient biopsies for prediction of clinical outcomes in patients with esophageal cancer has not yet been reported. Herein we describe a pilot prospective observational study with the goal of determining whether esophageal cancer PDOs created from treatment naive patients can model or predict clinical outcomes. Endoscopic biopsies of treatment-naive patients at a single tertiary care center were used to generate esophageal cancer PDOs, which were treated with standard-of-care chemotherapy, gamma-irradiation, and newer non-standard approaches, such as proton beam therapy or two small molecule inhibitors. Clinical outcomes of patients following neoadjuvant treatment were compared to their in vitro PDO responses, demonstrating the PDO’s ability to mirror clinical response, suggesting the value of PDOs in prediction of clinical response to new therapeutic approaches. Future prospective clinical trials should test the use of pre-treatment PDOs to identify specific, targeted therapies for individual patients with esophageal adenocarcinoma.

## Introduction

Esophageal cancer is a leading cause of cancer death worldwide^1^, with adenocarcinoma (EAC) being the most common subtype in the Western world^2^. Known risk factors for EAC include obesity, smoking, male gender, and gastro-esophageal reflux disease^3–6^; however, its incidence has continued to rise in the past 30 years. Despite the use of multimodal treatment strategies, including surgery, chemotherapy, radiation, and, more recently, immunotherapy, five-year survival remains below 20%^7^. Therefore, new approaches are required for understanding the molecular pathogenesis of esophageal cancer and identification of treatment approaches best suited for individual patients. In 2011, building on a previously developed murine model^8^, Hans Clevers and colleagues introduced human 3D gastrointestinal organoids, a novel in vitro culture system, which faithfully captures the structure and function of the native epithelial tissue^9^. To date, 3D organoids have been used to model disease and development affecting most organs, including esophagus^8–10^. Recently, publications emerged reporting the use of cancer organoids to model patients’ treatment responses in colorectal and pancreatic adenocarcinoma^11,12^. These studies, however, have been limited in their ability to prospectively predict treatment response to allow patient-derived organoids to be used as a clinical decision tool for the care of patients. In this study, we have sought to expand on the use of EAC PDOs as a predictive tool to model individual patients’ tumor responses in a prospective manner. Specifically, we have developed a protocol to establish and expand 3D esophageal cancer organoids from diagnostic biopsies collected from treatment-naive patients at the time of upper endoscopy. We then conducted studies on these organoids in parallel to treatments offered to patients, allowing these organoids to serve as treatment avatars. We also exposed these organoids to novel therapies not yet used for esophageal cancer patients and that we have identified previously^13^ and were able to demonstrate treatment efficacy in a pre-clinical setting.

## Materials and methods

### Tissue procurement

The study was approved by University of Pennsylvania Institutional Review Board (IRB protocol #813841) and informed consent obtained for biopsy specimens from patients undergoing diagnostic endoscopy for suspected esophageal cancer. All methods were performed in accordance with the University of Pennsylvania IRB committee's regulations on human subject research. All procedures were performed at the Hospital of the University of Pennsylvania. Tissue fragments were immersed in sterile PBS (Invitrogen) and immediately placed on ice.

### Patient-derived organoid culture and treatments

PDO lines were established as described previously^14^. Briefly, the tissue was digested in 1 mL Dispase (Corning, diluted 1:5 in HBSS), following by 1 mL Trypsin–EDTA (Invitrogen, 0.25%), followed by mechanical dissociation and passing through a 100 μm cell strainer (Falcon). The enzymes were inactivated by soybean trypsin inhibitor (Sigma-Aldrich), cells were washed in PBS, counted, and seeded at 5000 cells per 50μL Matrigel (Corning) per one well of a 24-well plate.

Cisplatin (Santa-Cruz) solution (1 mg/mL in PBS) was prepared fresh for each use; paclitaxel, stattic, and trametinib (Selleck Chemicals) solutions were prepared in DMSO at 10 mM, 100 mM and 100 μM, respectively, and stored at -80 °C. Organoids were subjected to γ-irradiation in Gammacell 40 Cesium 137 Irradiation Unit. An IBA Proteus Plus with a C230 cyclotron (Louvain-La-Neuve, Belgium) was used to deliver a uniform proton dose with a pencil beam scanned proton beam with range 10.5 cm in water and a spread-out Bragg peak width of 5 cm. Samples were placed in the middle of the spread-out Bragg peak. Radiobiological effectiveness (factor of 1.0) was accounted for in the total dose delivered for each proton irradiation.

### Imaging

For unbiased assessment of treatment efficacy, bright-field imaging and quantification of organoids was performed using Celigo Image Cytometer (Nexcelom) and its analytical algorithm (Fig. S1). The number of organoids in treated groups was normalized to vehicle-treated control group (relative PDO density). Live organoids were observed and imaged in bright-field mode using EVOS FL Cell Imaging System (Thermo Fisher Scientific). Hematoxylin–Eosin sections were imaged on Keyence BZ-X800 Cell Imaging Microscope.

### RNA sequencing

Organoid cultures were harvested after 48 h of treatment by mechanically dislodging the Matrigel™ with a P1000 tip and washed off with PBS. The organoids were spun down to remove supernatants and flash-frozen for shipping to GeneWiz, where RNA was isolated and sequenced using NEBNext Ultra RNA library prep with rRNA depletion, Agilent RNA ScreenTape, and Illumina HiSeq platform. The data quality was assessed using FastQC, paired-end reads were aligned to the human reference genome using the STAR 2-pass method. DESeq2 method was used to identify differentially expressed genes, which were then analyzed using Ingenuity Pathway Analysis (IPA) to identify canonical pathways. To ensure rigor and reproducibility, a colleague not involved in this study analyzed the data.

### Statistical analysis

All statistical analyses were conducted in GraphPad Prism 8. Two-way ANOVA with Tukey’s multiple comparisons test was used to determine whether organoid lines responded differentially to a particular treatment (Figs. 3, 6; *P*_i_ < 0.05 was considered significantly differential response).

## Results

### Patient-derived organoids from esophageal cancer biopsies

**Figure 1:**
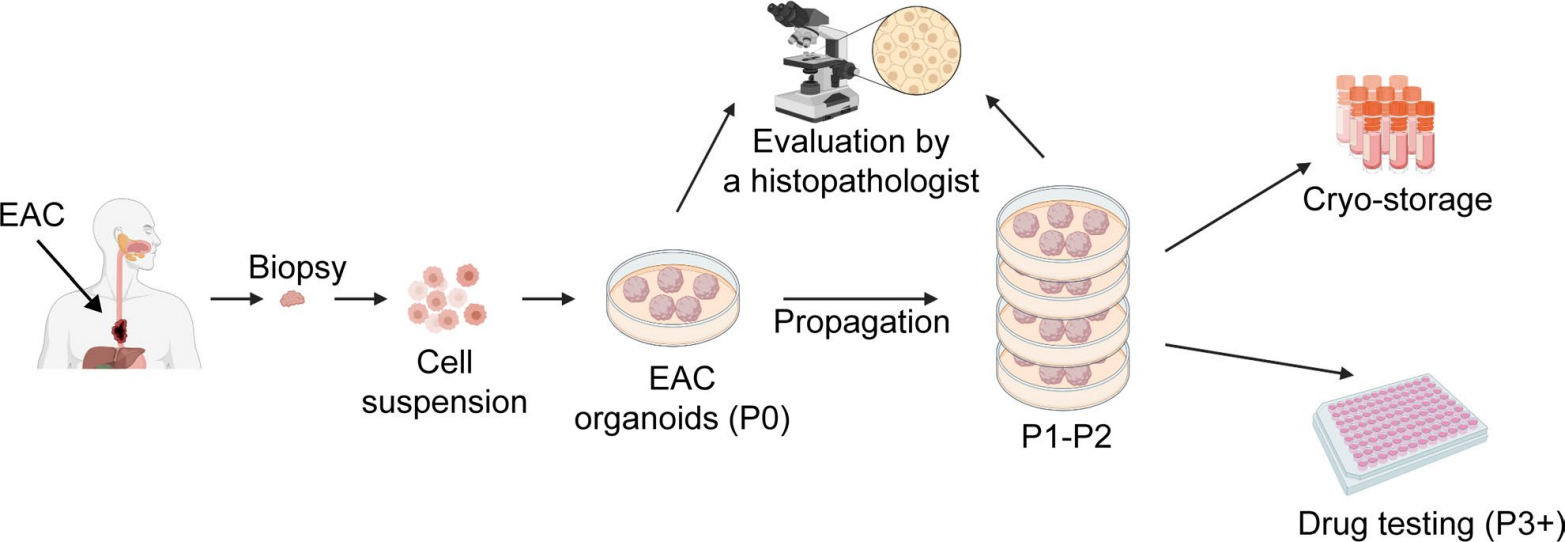
Schematic for EAC PDO generation. Diagnostic biopsies are enzymatically and mechanically dissociated into a single cell suspension, which is embedded in a basement membrane extract drop and grown in specialized culture medium. The PDOs are characterized by a pathologist to confirm EAC histology. Once sufficiently expanded and cryopreserved, the lines can be used for drug screening assays. Created with BioRender.com.

Tissue was collected from 9 patients undergoing esophagogastroduodenoscopy (EGD) for suspected esophageal cancer. Eight of these patients were treatment-naïve at the time of biopsy, and PDOs were successfully established from seven of these patients (Fig. 1). Three patients were subsequently excluded from further analysis because they did not receive neoadjuvant chemoradiation followed by surgery (one received palliative care, one received an esophagectomy only and one had not started treatment at the time of our studies). Thus, four EAC PDO lines were used in subsequent studies (Table 1). Of these patients, all had locoregional disease with no distant metastases and were deemed appropriate for neoadjuvant chemoradiation prior to esophagectomy. Pre-treatment histology demonstrated esophageal adenocarcinoma with one patient having the additional histological finding of signet cell features. All patients underwent post-neoadjuvant treatment positron emission tomography and computer tomography (PET/CT) scan prior to surgical resection. Patients were deemed to have a partial response (PR) if there was any change in tumor size or FDG-uptake on post-treatment PET/CT scan. This was termed clinical response after treatment prior to surgical resection. Surgical resection occurred at least 6 weeks after completion of neoadjuvant treatment. Surgical tissue was evaluated by an anatomic pathologist at the time of resection and was described as a partial response if there was residual tumor with treatment effect noted. This was termed pathologic response post treatment. No tumors were described as having a complete response.

**Table 1:** Patient characteristics.

PDO line	EACorg000	EACorg002	EACorg006	EACorg011
Patient No	134	153	155	168
Age	76	70	79	63
Gender	M	M	M	M
Pre-treatment Stage	T2N0M0	T3N1Mx	T3N2M0	T3N1M0
Histology	Adenocarcinoma	Adenocarcinoma with signet cell features	Adenocarcinoma	Adenocarcinoma
Differentiation	Moderate	Poor	Moderate	Poor
Neoadjuvant Treatment	SC	FOLFOX + PBT	SC	SC
Clinical response	NR	PR	PR	PR
Pathological response	PR	PR	PR	PR
Post-treatment stage (pathological)	T1bN0M0	T3N0M0	T2N2M0	T3N0M0

*SC* standard of care, platinum-based doublet with paclitaxel and radiation therapy, *PBT* proton beam therapy, *PR/NR* partial response/no response, *FOLFOX* Folinic acid-5-Fluorouracil-Oxaliplatin.

### EAC PDOs can serve as treatment avatars

**Figure 2:**
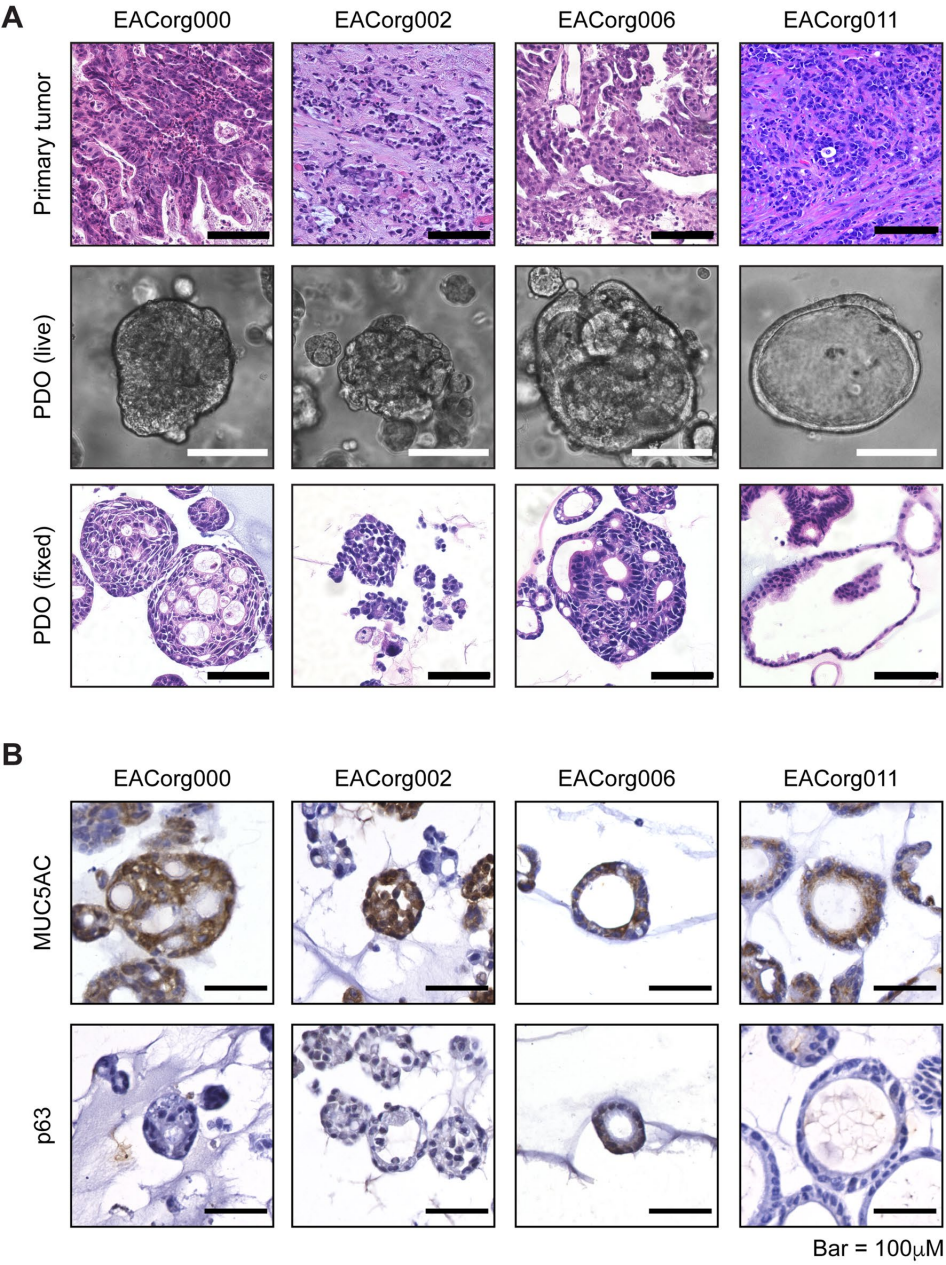
EAC PDOs recapitulate histological features of original tumors. (**A**) Histological features of EAC PDO lines and primary tumors from which they were derived, presented as bright-field images of hematoxylin–eosin stained sections (scale bar = 100 μm). (**B**) Positive cytoplasmic staining for EAC marker MUC5AC and negative nuclear staining for ESCC marker p63 in EAC PDOs.

**Figure 3:**
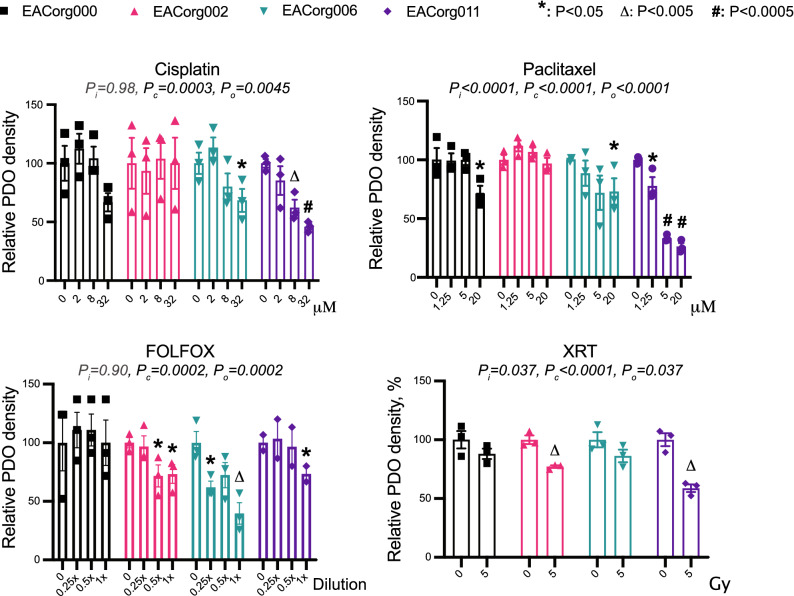
EAC PDOs serve as avatars for patients’ responses to therapy. Relative PDO density in response to components of standard of care and alternative treatments. 2-way ANOVA parameters: P_i_ = significance of interaction (differential responses of organoid lines), P_c_ = significance of concentration/dose, P_o_ = significance of organoid line. Error bars = SEM, *P < 0.05, Δ*P* < 0.005, #*P* < 0.0005.

The PDOs recapitulated the histopathological characteristics of the original tumors, as validated by a blinded clinical pathologist (Fig. 2A). Furthermore, all EAC PDOs stained positive for the EAC marker MUC5AC and negative for the ESCC marker p63^15^ (Fig. 2B). We treated these PDOs with several standard-of-care neoadjuvant therapies, including cisplatin, paclitaxel, γ-irradiation, and FOLFOX (Table 1). The PDOs responded differentially to the treatments (Fig. 3, S2) and mimicked the patients’ clinical/pathological responses to analogous regimens. EACorg000 and EACorg006 responded to both cisplatin and paclitaxel, but not to γ-irradiation; EACorg002 responded to FOLFOX and γ-radiation, but not to cisplatin or paclitaxel. Interestingly, EACorg011 responded to all treatments.

### EAC PDOs and transcriptional analysis of response to chemotherapy

**Figure 4:**
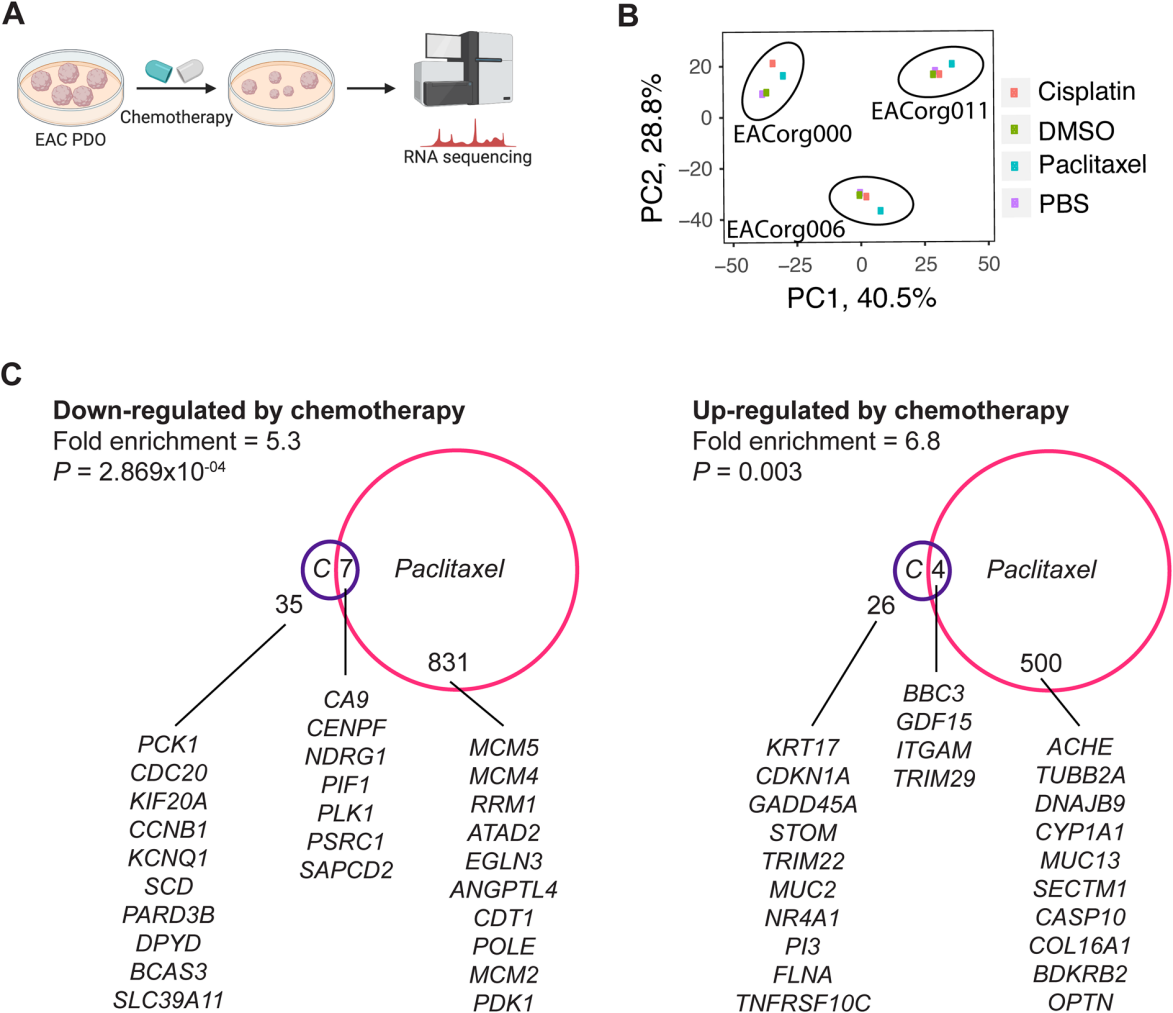
EAC PDOs can be used to identify drivers of therapeutic response. (**A**) Schematic of the RNAseq setup. Created with BioRender.com. (**B**) Principle component analysis (PCA) of differentially expressed genes with *P* < 0.02. (**C**) Venn diagrams of DEGs (*P* < 0.02) induced by cisplatin or paclitaxel treatments, and the 10 most significantly altered genes are listed. Significance of overlap and fold enrichment as determined by hypergeometric test.

To identify potential drivers of resistance to chemotherapy, we conducted RNA sequencing on three PDO lines treated either with chemotherapy (cisplatin/paclitaxel) or vehicle control (PBS/DMSO, respectively) (Fig. 4A). Interestingly, principal component analysis (PCA) revealed the samples clustered based upon the organoid line, and not based on the type of treatment (Fig. 4B).

**Figure 5:**
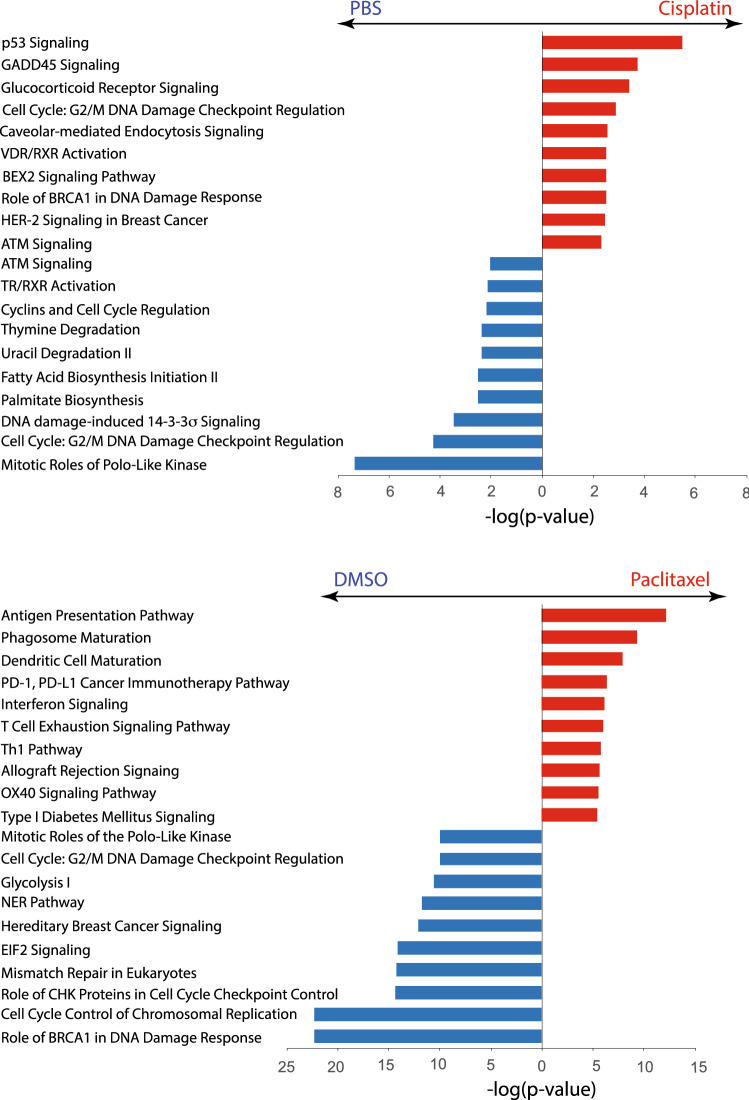
Canonical pathways affected by cisplatin or paclitaxel treatment. Top 10 differentially affected canonical pathways identified by Ingenuity pathway analysis are listed according to their p-value (-log). Red bars represent pathways enriched in chemotherapy-treated PDO, blue bars represent pathways enriched in vehicle-treated PDO.

We compared the differentially expressed genes (DEGs) induced by cisplatin (Table S1A) or paclitaxel (Table S1B) across the three PDO lines tested and found significant upregulation of 26 and 500 genes, respectively. Strikingly, only four genes (*BBC3, GDF15, ITGAM* and *TRIM29*) were upregulated in response to both treatments. Similarly, 35 and 831 genes were downregulated in response to cisplatin or paclitaxel, respectively, but only seven genes (*CA9, CENPF, NDRG1, PIF1, PLK1, PSRC1* and *SAPCD2*) appeared on both lists (Fig. 4C). This implies that cisplatin and paclitaxel induce different changes in gene expression. We also conducted Ingenuity Pathway Analysis (IPA) on the DEG lists, and the most significantly affected pathways are presented in Fig. 5, Table S2A–D. Here, the differences between the effects of cisplatin and paclitaxel were further underscored: while cisplatin affected the pathways related to cell cycle and DNA damage, paclitaxel affected immunity-related pathways (20% and of 45% pathways in Fig. 4, respectively).

Since EACorg011, one of the lines used in RNAseq, was generated from an on-treatment biopsy, we confirmed that its inclusion did not skew the analysis. Indeed, fold-change values obtained by assessing gene expression changes in EACorg000 + EACorg006 + EACorg011 aligned with fold-change values obtained from EACorg000 + EACorg006 with R^2^ > 0.7 (Fig. S3).

### Esophageal cancer PDOs can be used for testing of novel therapies

**Figure 6:**
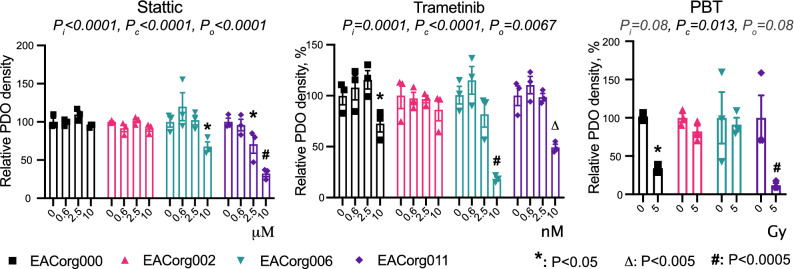
EAC PDOs can be used to identify susceptibilities to new therapeutics. Relative PDO density in response to experimental treatment. 2-way ANOVA parameters: P_i_ = significance of interaction (differential response of organoid lines), P_c_ = significance of concentration/dose, P_o_ = significance of organoid line. Error bars = SEM, *P < 0.05, Δ*P* < 0.005, #*P* < 0.0005.

We next tested Stattic (STAT3 inhibitor) and Trametinib (MEK1/2 inhibitor), two agents that we have previously identified in preclinical models as promising therapies for esophageal cancer^13^, as well as proton beam therapy (PBT), which is currently in trials for treatment of EAC, in the PDO lines (Fig. 6, S4). EACorg000 responded to PBT and Trametinib. EACorg006 responded to Stattic and Trametinib. EACorg002 did not respond to any treatments, whereas EACorg011 was susceptible to all treatments.

## Discussion

We generated PDOs from patients with EAC with a success rate of 78%. Four of these organoid lines were used in this pilot study, and all demonstrated treatment responses nearly identical to the patients from whom they were derived. For example, EACorg000 was less responsive to drug treatments than EACorg006, which is consistent with the lack of clinical response by PET/CT for patient 134 and partial clinical response for patient 155, whereas by final pathology reports, both patients responded partially, similar to their respective organoid data (Table 1). This observation suggests that a PDO’s response to therapy may be a useful indicator of the degree of patient tumor response to the same treatment, when used in parallel to clinical treatment, and may be a useful adjunct to a PET/CT scan.

Patient 153 had EAC with signet cell features. Such tumors have poor response to standard treatment regimens^16^, and accordingly, patient 153 received FOLFOX in combination with proton beam therapy, which resulted in a clinical and pathological partial response (Table S1). Importantly, EACorg002 (derived from patient 153) did not respond to cisplatin and paclitaxel, while FOLFOX led to reduced PDO density. Interestingly, EACorg002 responded to γ-irradiation, but not to PBT (Figs. 3, 6). These findings are interesting, because there are few studies directly comparing the efficacy of PBT to γ-irradiation in EAC, with ongoing clinical trials. In a recent randomized phase IIB trial of Proton Beam Therapy versus Intensity-Modulated Radiation Therapy (IMRT) at 50.4 Gy for locally advanced esophageal cancer, PBT was identified to have similar progression free survival (PFS) as IMRT with reduced risk and severity of adverse events^17^. Our results suggest that response to PBT versus IMRT may be patient-specific, and PDOs provide for new opportunities to study differences between both types of radiotherapy treatment on an individualized basis.

While several clinical trials are ongoing, the role for adjuvant therapy in patients with residual disease following induction chemoradiation followed by surgery is currently limited, in part under the assumption that residual or recurrent tumors may be less chemoresponsive. However, in our study, EACorg011 remained sensitive to all types of therapy, despite being derived from a post-treatment patient biopsy. This observation suggests that organoid-based therapy screening models might also inform post-surgical adjuvant protocols, and thus have the potential to improve treatment options for residual disease.

RNAseq revealed that even after exposing PDOs to chemotherapy, they maintained a similar gene expression profile to pre-treatment organoids, as evident from the PCA (Fig. 4B). Also, expression profiles of the different PDO lines had little overlap, demonstrating the heterogeneity of EAC. Further investigation demonstrated little overlap in the gene expression profiles of organoids treated with cisplatin versus paclitaxel. To our knowledge, we are the first to characterize these pre- and post-treatment effects of standard of care chemotherapy agents in EAC.

Our data underscore the heterogeneity that exists in esophageal cancer as a disease. This heterogeneity necessitates an individualized approach to therapy. We have demonstrated the use of patient derived pre-treatment esophageal cancer organoids from biopsies as a viable approach. The robustness of this protocol also lends it to be an ideal system for trialing novel therapies in a pre-clinical setting and should be considered for use instead of traditional 2D cell lines, which do not capture the intratumoral and intertumoral heterogeneity of esophageal cancer, and in tandem with genetically engineered mouse models. Future studies should focus on improving the PDO culture system to include components of the tumor microenvironment, which are now important therapeutic targets^18^. In the recently published Phase III Keynote-181 study of Pembrolizumab versus Chemotherapy in Advanced Esophageal Cancer, 628 patients with advanced/metastatic squamous cell carcinoma or adenocarcinoma of the esophagus, who already progressed on prior therapy, were randomized to pembrolizumab or investigator’s choice of chemotherapy (including paclitaxel, docetaxel, or irinotecan)^18^. Pembrolizumab prolonged overall survival, compared to chemotherapy, as a second-line therapy for advanced esophageal cancer in patients with a PDL-1 CPS ≥ 10 (Combined Positive Score), with fewer treatment-related adverse events. It is unclear, however, based on this study, if other tumor intrinsic factors might be able to predict treatment efficacy, independent of PDL-1 status. For example, others have found that PDOs predicted positive treatment response to targeted therapies independent of tumor intrinsic status (i.e. Her2neu status)^19^.

In summary, in this pilot study, we have demonstrated that the prospective use of PDOs may offer relevant clinical insights and have the potential to predict response to chemoradiation therapy and thus inform treatment selection. The robustness of this protocol also lends it as an ideal system for testing novel therapies that could be considered in tandem with standard of care in the clinical arena.

## Supplementary Information

Supplementary Information 1.

Supplementary Information 2.

Supplementary Information 3.
