# Supplementary material for: Patient-derived organoids as a platform for modeling a patient’s response to chemoradiotherapy in esophageal cancer
Source: Sci Rep. 2021 Oct 29;11:21304. doi: 10.1038/s41598-021-00706-8 (PMC8556341; doi:10.1038/s41598-021-00706-8)
Supplement: Supplementary file 1 — Supplementary Information 1. [file 41598_2021_706_MOESM1_ESM.pdf]

Supplemental figures

Supplementary Figure 1

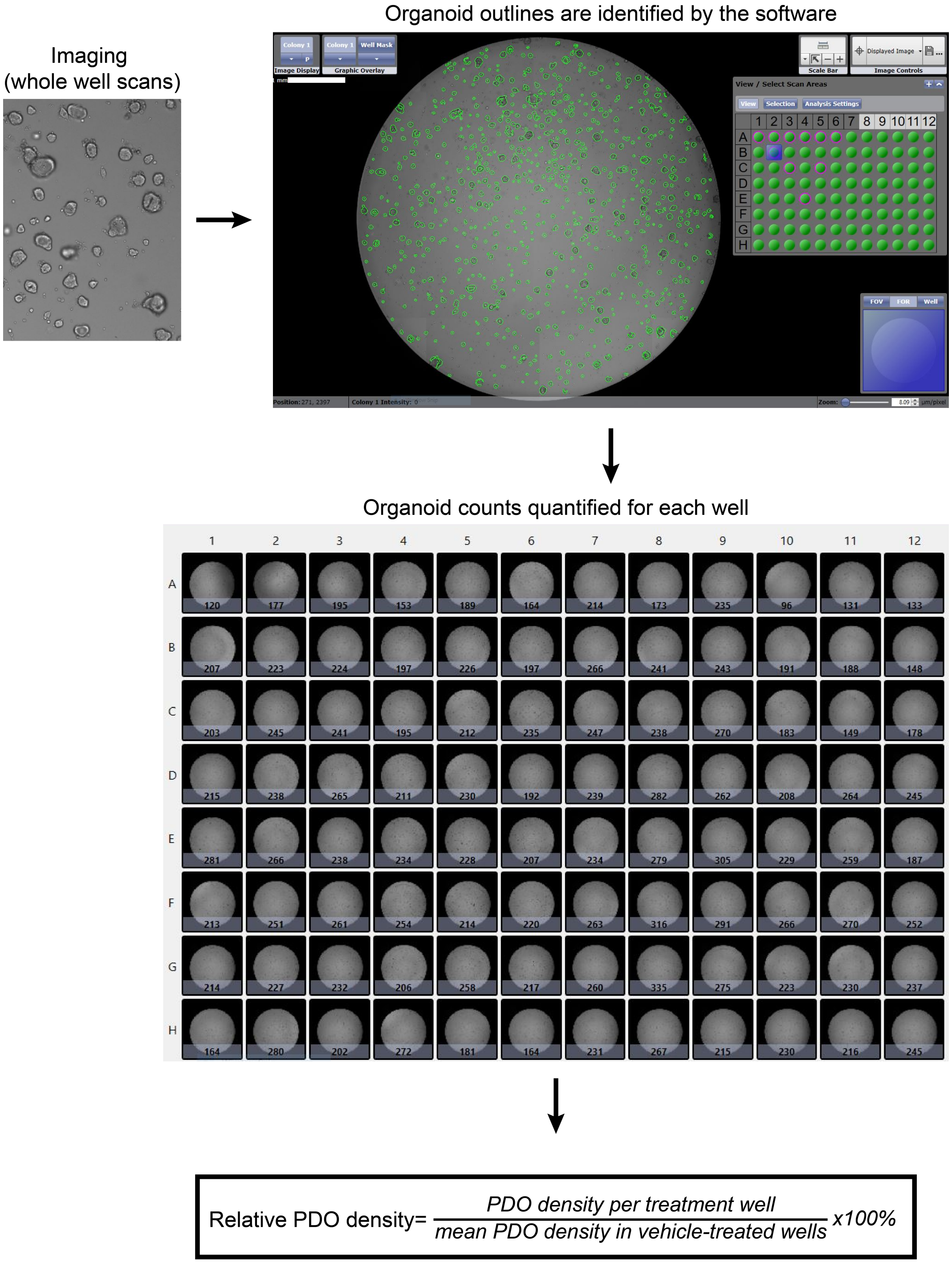

Supplementary Figure 1: Schematic of PDO density quantification using Celigo Image Cytometer Software.

Supplementary Figure 2

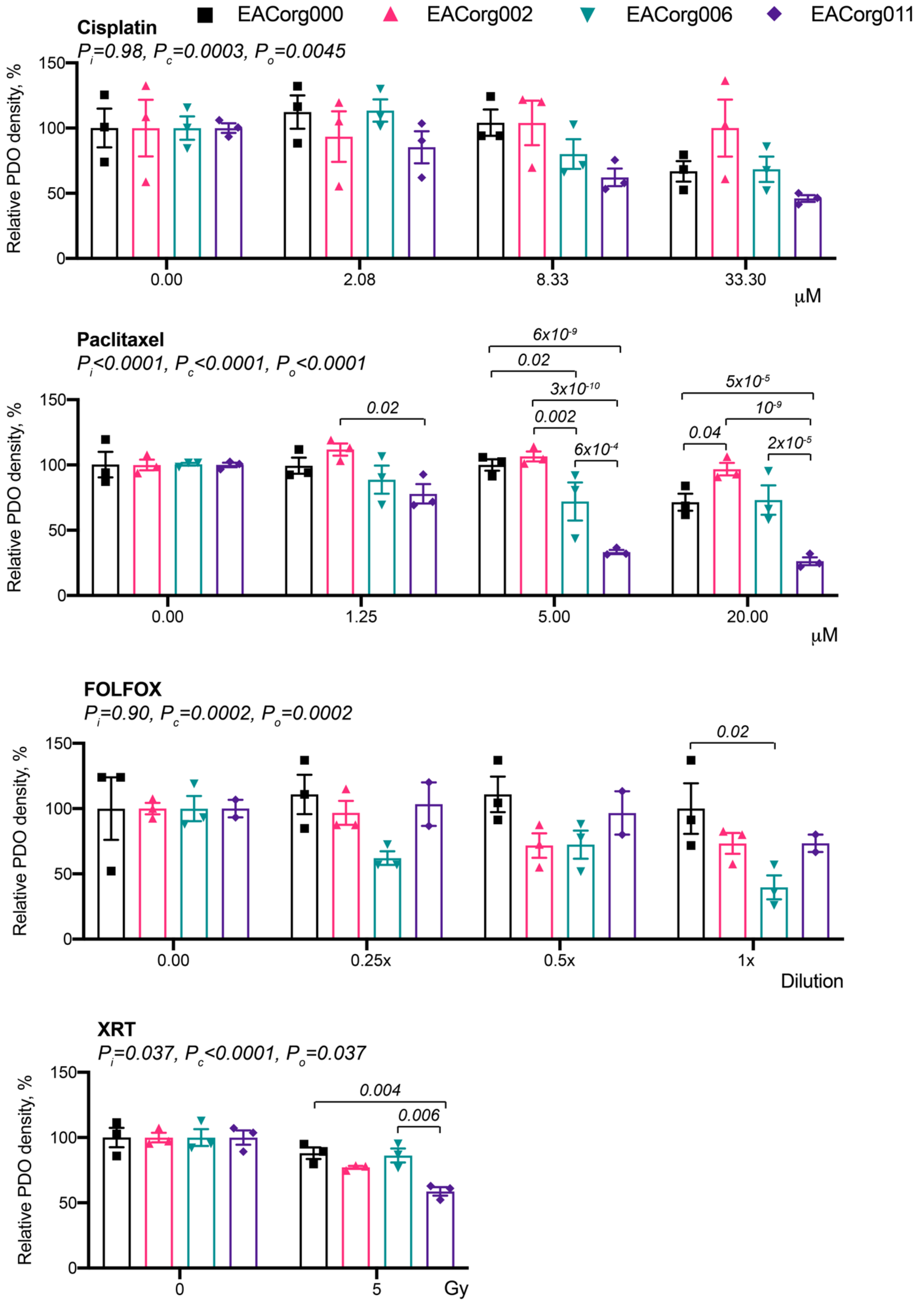

Supplementary Figure 2: EAC PDOs serve as avatars for patients' responses to therapy. Relative PDO density in response to components of standard of care and alternative treatment. 2-way ANOVA parameters: P_i=significance of interaction (differential response of organoid lines), P_c=significance of concentration/dose, P_o=significance of organoid line. Error bars = SEM, significant P-values for multiple comparisons are listed on the graphs.

Supplementary Figure 3

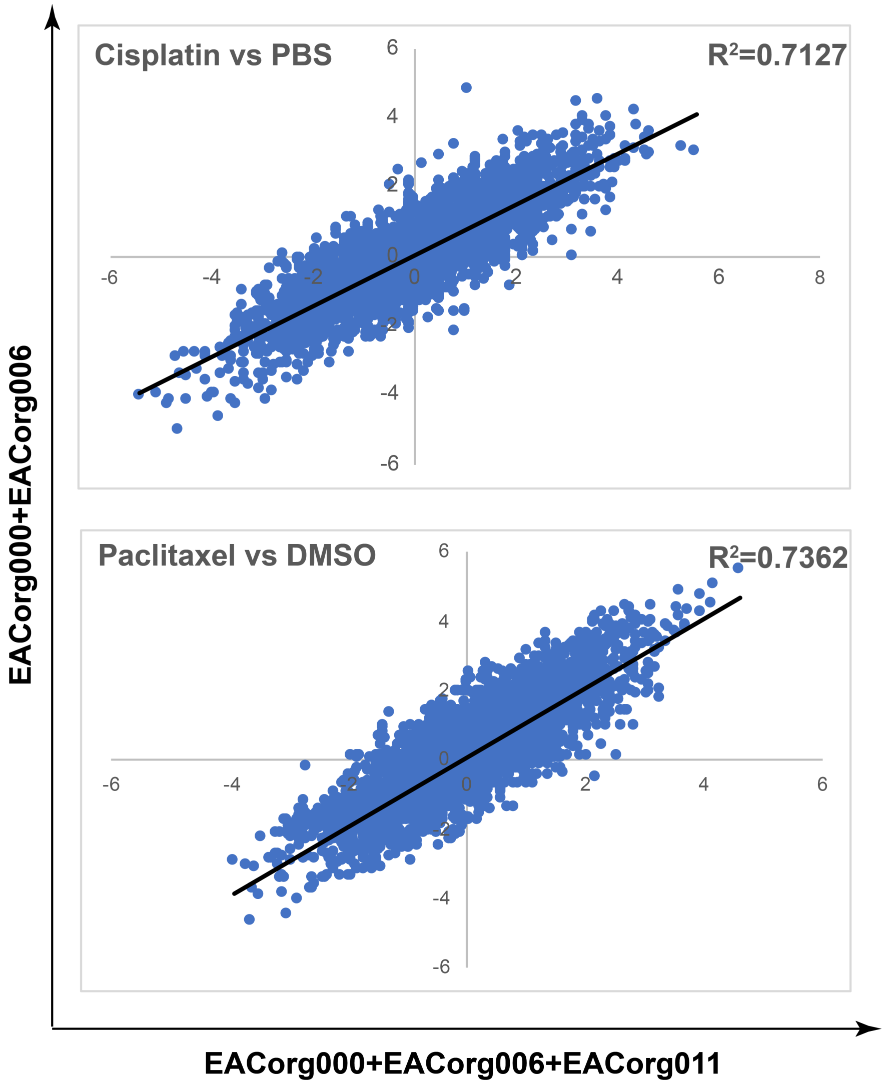

Supplementary Figure 3: Introduction of EACorg011 does not substantially alter the DEG lists. DEG lists obtained by analyzing datasets comprised by EACorg000+EACorg006+EACorg001 were plotted against the DEG lists obtained by analyzing the EACorg000+EACorg006 datasets. R^2 values for linear relationship displayed on the graphs.

Supplementary Figure 4

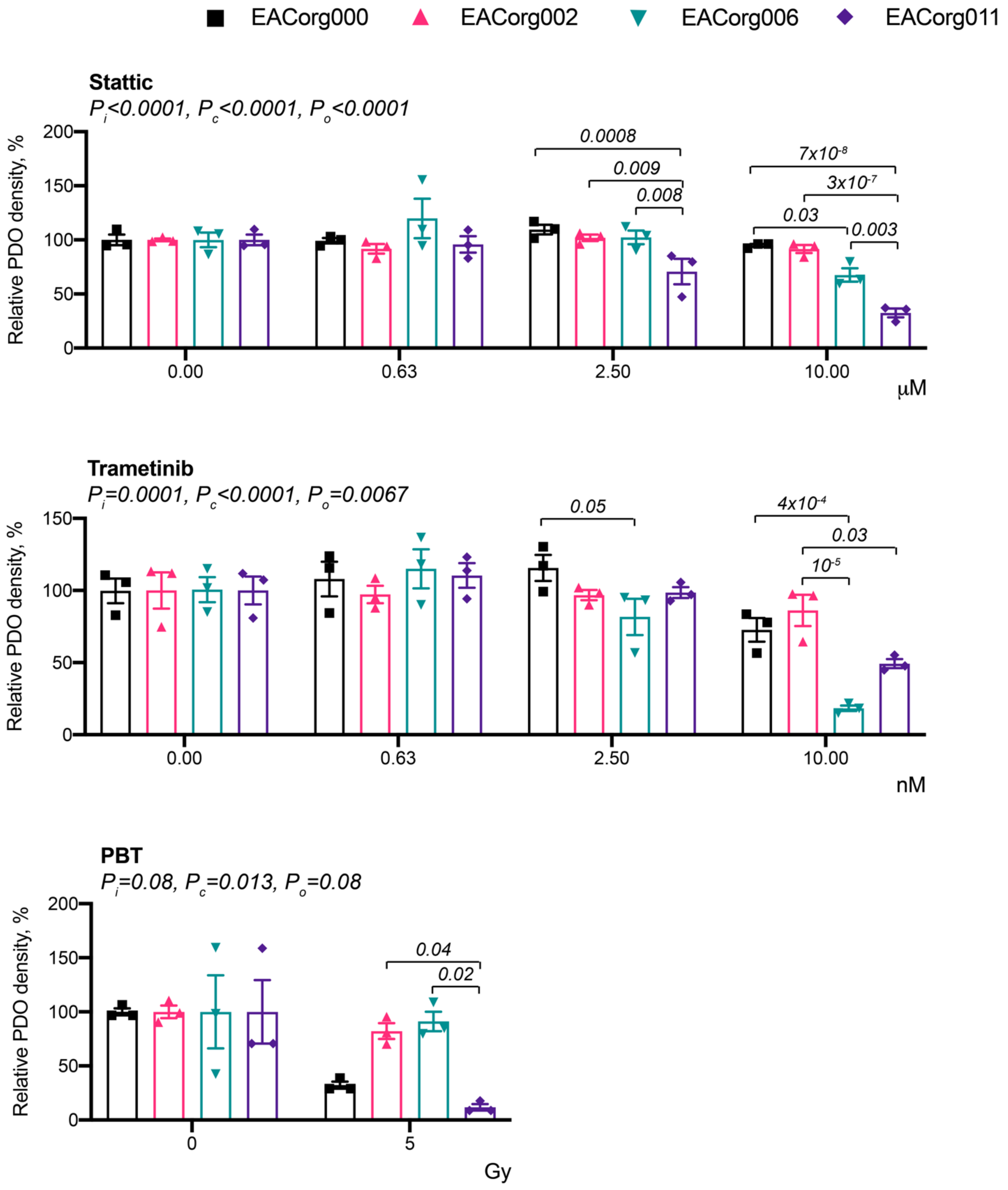

Supplementary Figure 4: EAC PDOs respond differentially to experimental therapeutics. Relative PDO density in response to components of standard of care and alternative treatment. 2-way ANOVA parameters: P_i=significance of interaction (differential response of organoid lines), P_c=significance of concentration/dose, P_o=significance of organoid line. Error bars = SEM, significant P-values for multiple comparisons are listed on the graphs.
